# Brownian motion studies of viscoelastic colloidal gels by rotational single particle tracking

**DOI:** 10.1107/S2052252514006022

**Published:** 2014-04-14

**Authors:** Mengning Liang, Ross Harder, Ian K. Robinson

**Affiliations:** a Department of Physics, University of Illinois at Urbana-Champaign, Urbana, IL 61801, USA; b Center for Free-Electron Laser Science, Deutsches Elektronensynchrotron, Notkestrasse 85, 22607 Hamburg, Germany; c Argonne National Lab, Argonne, IL 60439, USA; d Centre for Nanotechnology, University College, London WC1H 0AH, England

**Keywords:** rotational X-ray tracking, rotational dynamics, colloidal gels

## Abstract

Colloidal gels have unique properties due to a complex microstructure which forms into an extended network. Although the bulk properties of colloidal gels have been studied, there has been difficulty correlating those properties with individual colloidal dynamics on the microscale due to the very high viscosity and elasticity of the material. We utilize rotational X-ray tracking (RXT) to investigate the rotational motion of component crystalline colloidal particles in a colloidal gel of alumina and decanoic acid. Our investigation has determined that the high elasticity of the bulk is echoed by a high elasticity experienced by individual colloidal particles themselves but also finds an unexpected high degree of rotational diffusion, indicating a large degree of freedom in the rotational motion of individual colloids even within a tightly bound system.

## Abstract 2

The method of rotational X-ray tracking is used to probe the microradian rotational motion of individual crystalline colloids in a colloidal gel to determine the viscous and elastic properties.

## 1. Introduction   

Complex materials such as colloidal gels have long been of interest for their unique properties and potential industrial applications (Lin *et al.*, 1989 ▶; Lu *et al.*, 2008 ▶; Shih *et al.*, 1990 ▶; Pickrahn *et al.*, 2010 ▶). Colloidal gels form when inter-particle forces, either native to the colloidal particles, or through an intermediary such as a bridging polymer, are prevalent enough to form wide spanning structures that largely determine the properties of the system (Lin *et al.*, 1989 ▶; Lu *et al.*, 2008 ▶; Shih *et al.*, 1990 ▶; Pickrahn *et al.*, 2010 ▶). Challenges in determining the properties of colloidal gels are due to the microstructuring of the material and high viscoelasticity. Macroscopic properties such as the elastic and viscous components of colloidal gels can be measured with standard rheological techniques but investigating the microscopic properties remains challenging (Shih *et al.*, 1990 ▶; Pickrahn *et al.*, 2010 ▶; Schenker *et al.*, 2008 ▶; Gao & Kilfoil, 2007 ▶; Dibble *et al.*, 2006 ▶; Guo *et al.*, 2011 ▶; Raghavan *et al.*, 2000 ▶). Micro-structure can and has been extensively studied (Lu *et al.*, 2008 ▶; Shih *et al.*, 1990 ▶; Pickrahn *et al.*, 2010 ▶; Schenker *et al.*, 2008 ▶; Gao & Kilfoil, 2007 ▶; Dibble *et al.*, 2006 ▶). Techniques such as confocal microscopy and dynamic light scattering are used to probe the dynamics of colloidal particles in a colloidal gel with a resolution limit of a few hundred nanometers (Pickrahn *et al.*, 2010 ▶; Schenker *et al.*, 2008 ▶; Gao & Kilfoil, 2007 ▶; Dibble *et al.*, 2006 ▶; Guo *et al.*, 2011 ▶). Studies have seen dynamic heterogeneity and bimodal populations of freely diffusing particles which correspond to particles not comprising the gel scaffold and arrested particles, those particles which participate in the gel and most importantly determine the macroscopic properties (Pickrahn *et al.*, 2010 ▶; Schenker *et al.*, 2008 ▶; Gao & Kilfoil, 2007 ▶; Dibble *et al.*, 2006 ▶; Guo *et al.*, 2011 ▶). An increase in the number of arrested or stationary colloids, or a decreased mobility of the colloidal particles, has been correlated with an increase to the high viscoelasticity characteristic of colloidal gels (Shih *et al.*, 1990 ▶; Pickrahn *et al.*, 2010 ▶; Schenker *et al.*, 2008 ▶; Gao & Kilfoil, 2007 ▶; Dibble *et al.*, 2006 ▶; Guo *et al.*, 2011 ▶). A typical colloidal gel has viscous and elastic components that would limit the translational motion of a micron scale particle to an Å or less, far below the resolution of any but the best electron microscopes, so the classification of the particles as ‘stationary’ is justified. As such, there is still no direct link between the motion of an individual colloidal particle which forms this network and the viscoelastic response of the bulk material. Active driving of the colloidal particles which can be used to probe other highly viscoelastic materials would not be suitable as forceful disruption of the network would result in destruction of the exact network that one is trying to probe. Thus, a passive method of determining particle motion in a colloidal gel is necessary. In this work we probe the motion of those particles which have been classified as ‘stationary’ and ‘arrested’.

One strategy is to move from translational motion to rotational motion which is inherently more sensitive to forces in viscous media because it has cubic dependence on size, as opposed to linear for translational forces. In addition, unless there is site-specific binding in a colloidal gel, rotational motion does not compromise the structural integrity of the gel. Many innovative techniques to determine rotational motion have been developed recently, but there are still limitations. Video microscopy techniques that track rotational motion of single particles require anisotropy, whether optical (Anker & Kopelman, 2003 ▶) or physical (Fakhri *et al.*, 2010 ▶; Li *et al.*, 2011 ▶). Ensemble methods such as depolarized dynamic light scattering or fluorescence correlation spectroscopy also require optical or physical anisotropy (Berne & Pecora, 1975 ▶; Pieper & Enderlein, 2011 ▶; Rogers *et al.*, 2012 ▶). NMR can measure the rotation of isotropic ensembles but only at longer timescales (Esteve *et al.*, 1984 ▶). The resolution of most optical techniques is of the order of 1°, still far larger than the rotational motion of particles in a colloidal gel. X-ray Bragg diffraction has been used to study the rotational motion of crystals attached to a protein to determine protein properties (Sasaki *et al.*, 2000 ▶; Shimizu *et al.*, 2008 ▶). The motion observed for this tethered particle reflects the coupled translational and rotational motion of the corresponding free system. Rotational X-ray tracking (RXT) observes the pure rotational motion of free crystals at the microradian scale, an increase of three orders of magnitude from standard optical techniques. Single particle rotational tracking by X-ray diffraction was explored systematically for a number of systems (Liang, 2008 ▶) and observed with XPCS studies of carbon-black (Shinohara *et al.*, 2013 ▶). In this work we substantiate the use of RXT as a way to measure viscoelasticity and investigate the motion of gels of alumina micro- and nanocrystals, which are currently used in abrasives, polishing and advanced ceramics processing and have been the subject of much study (Bell *et al.*, 2005 ▶; Studart *et al.*, 2006 ▶; Bergstrom *et al.*, 1992 ▶).

## 2. Method   

Rotational X-ray tracking can observe the rotational behaviors of any crystal which has observable Bragg diffraction, from protein crystals to multilayers, in addition to commonly considered crystals. Similar to traditional translational Brownian motion, the rotational motion of nanocrystals embedded in a medium can act as a probe, coupling to the characteristics, motions and phase transitions of the medium itself and the interaction between the particle and medium. In our case of a colloidal gel the colloid acts not merely as a probe, but its motion is a direct measure of the properties of the gel network that it comprises.

X-rays can image optically opaque materials and Bragg scattering can differentiate between crystals of different sizes and lattices. In applying RXT to both solid and liquid phase alumina mixtures, we have observed a number of behaviors – diffusive, convective, stochastic and drift. Further investigation has discovered the nanoscale motion of materials such as the pigment particles of dried paints and powder laced epoxies, systems that are not commonly thought of as mobile.

**Figure 1:**
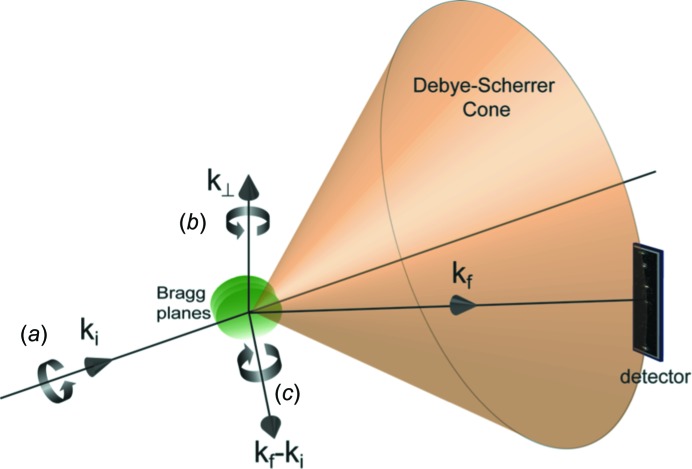
The Bragg scattering geometry of the experiment. **k**_i_ is the incident wavevector, **k**_f_ is the diffracted wavevector and **k**_f_ − **k**_i_ is the scattering vector. **k**_i_ and **k**_f_ define the scattering plane and **k**_⊥_ is the vector perpendicular to that plane. Rotation of the crystal around **k**_i_ (*a*) corresponds to motion of the Bragg peak around the Debye–Scherrer cone and rotation around **k**_⊥_ (*b*) corresponds to motion of the Bragg peak across the powder ring. Rotational around the scattering vector **k**_f_ − **k**_i_ (*c*) is not observable.

**Figure 2:**
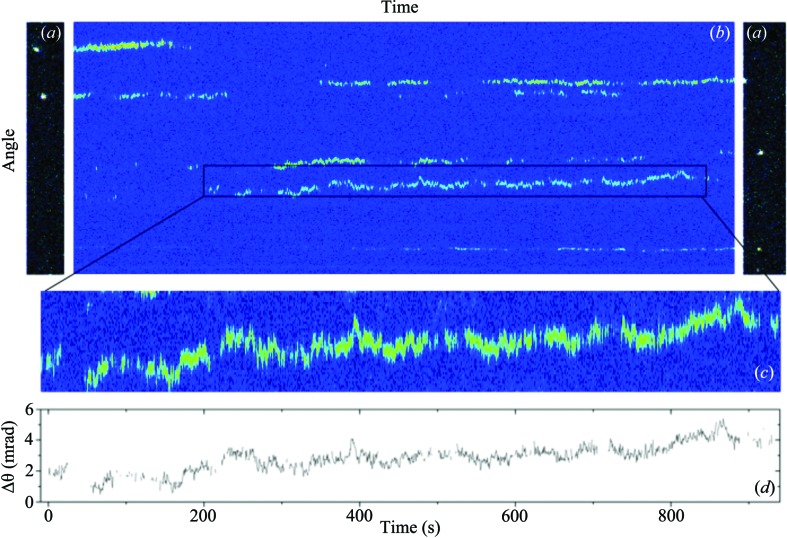
RXT determines very small angular displacements by using the diffraction intensity of individual crystals. (*a*) The first and last frames of the time series, showing the (104) alumina powder ring in the vertical direction. (*b*) Each of the powder ring intensity frames is radially integrated (horizontally) and plotted *versus* time. (*c*) Trajectories are extracted to obtain the angle *versus* time for a single particle. (*d*) The trajectory is then tracked to quantify the angular motion.

RXT does not depend upon an optical or particle shape anisotropy, but relies on the orientational marker inherent to a crystal, which is its lattice. Fig. 1 ▶ illustrates the scattering geometry and the axes of rotation for a crystal in Bragg geometry. The diffraction condition of a crystal is given by Bragg’s Law, 2*d*sinθ = λ, where *d* is lattice spacing, θ is Bragg angle and λ is wavelength. The diffracted intensity is precisely sensitive to the direction of the lattice planes, and the position of the Bragg reflection defines the orientation about 2 rotational axes, **k**_i_, the incident wavevector, and **k**_⊥_, the vector which is perpendicular to the scattering plane. Any change in the position of the diffracted intensity will indicate a rotation of the crystal. Rotation of the Bragg planes around **k**_i_ corresponds to motion of the Bragg peak around the Debye–Scherrer cone and rotation around **k**_⊥_ corresponds to motion of the Bragg peak across the powder ring dependent on the crystal size and energy resolution of the X-rays. Our technique is not sensitive to crystal rotation about the scattering vector **k**_f_ − **k**_i_. The theoretical time resolution depends on the size of the crystal and the photon flux. Enough photons must be scattered in the time step desired to accurately identify the position of a Bragg peak. Technologically, the angular and time limits are determined by the signal-to-noise ratio, pixel size and readout time of the detectors and will greatly benefit with advancing detector technology. The flux of modern X-ray sources allows for the tracking of particles as small as 100 nm, with an angular resolution of 8 µrad at 1 ms time resolution, with a standard detector, an increase of three orders of magnitude in angular resolution for a particle at the diffraction limit of visible light. Larger crystals would allow for better time resolution. Fig. 2 ▶ provides an example of the data obtained and the analysis procedure.

## 3. Experiment   

In order to test the veracity of RXT to probe material properties, a system of large alumina particles embedded in a standard Newtonian liquid, glycerol, >99% pure, was studied which can be compared with bulk rheometry data. Alumina crystals of median size 76 µm and 42 µm (Kramer Industries) were suspended in glycerol at a 30% volume fraction. The sample was measured in a capillary in transmission geometry at the X11 beamline at Doris III with an unfocused 15.2 keV beam and a flux of 10^13^ photons s^−1^ in a spot size of 1 × 1 mm. The beam was further attenuated to 4 × 10^12^ photons s^−1^ and 1 × 10^12^ photons s^−1^. Diffraction of the (104) Bragg reflection of alumina was measured with a Mythen strip detector with time steps of 30  and 50 msec at a sample-to-detector distance of 0.7 m. The same glycerol was studied with rheometry measurements performed with an ATS Rheosystems Stress­tech Rheometer in cone and plate geometry at the North Carolina State University Food Rheology Laboratory.

Having tested RXT for a standard, we investigate the colloidal gel formed by nanoscale alumina crystals in fatty acids using RXT, which are more challenging in terms of particle size and viscosity limits. As a solid, fatty acids form organic crystals (Baun, 1961 ▶) and as a liquid, nanoparticle alumina/decanoic suspensions have been seen, by traditional rheological measurements, to exhibit gelation, resulting in high viscoelasticity (Bell *et al.*, 2005 ▶).

Specifically, we observed the rotational motion of single-crystal 340 nm alumina suspended in decanoic acid (C_9_H_19_COOH) at a 40–50% volume fraction. The angular resolution of 82 µrad is determined by the 2 × 2 binning of the (20.5 µm)^2^ CCD detector pixels and the sample detector distance of 0.5 m. Higher resolution can be obtained by unbinning the detector and increasing the sample–detector distance, but was unnecessary for our sample.

The decanoic acid samples were prepared with alumina nanoparticles (Buehler Inc) at a 2.5% volume fraction. Diffraction from dry alumina shows stationary behavior (Fig. S1). The solution was sonicated in a warm water bath for > 3 h above the melting point of 31°C. Fatty acids adsorb onto the surface of alumina (Bell *et al.*, 2005 ▶), sterically stabilizing the particles and minimizing interface effects and particle aggregations, confirmed by SEM imaging (Fig. S2).

Sedimentation increases the concentration forming a gel. The colloid volume fraction in the volume probed by the beam was determined from the number of particles on the powder ring, the solid angle of the detector and the multiplicity of the (104) reflection of alumina. On average, 5 particles were seen on the detector, corresponding to a 40% volume fraction for the gel phase. A 3 μL droplet deposited on a hydrophobic octadecyltriethoxy­siloxane (OTE) coated silicon wafer was placed on a variable-temperature sample stage under rough vacuum. The sample was measured with a 5 circle diffractometer at sector 34ID-C at the Advanced Photon Source (APS). A focused 8, 9 or 11 keV beam, with a flux of the order of 10^9^ photons s^−1^, was incident on the droplet in transmission geometry. Flux was controlled by tapering the undulator and with attenuators. Diffraction of the (104) Bragg reflection of alumina single crystals was measured with a Princeton Instruments charge-coupled device (CCD) camera at temperatures above and below the melting point of decanoic acid. Time series were taken at intervals of 0.02  and 0.1 s to obtain angular trajectories.

## 4. Results   

**Figure 3:**
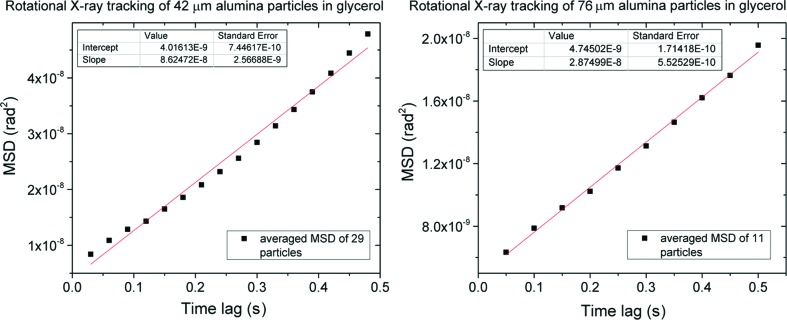
Mean-squared displacement (MSD) *versus* time of 42 µm and 76 µm alumina particles in glycerol.

Viscosity determination of glycerol by RXT was performed by tracking the rotational trajectories of 76 µm particles and 42 µm particles. They are fitted using standard Newtonian rotational diffusion, where the angular mean-squared displacement (MSD) Δ < θ(*t*)^2^ ≤ 2*D*_r_t (Fig. 3 ▶). The rotational diffusion coefficient is given by *D*_r_ = *k*_B_ *T*/*f*_r_, where *f*_r_ is the rotational drag coefficient of a sphere of radius *R*, given by *f*_r_ = 8πη*R* ^3^. Rheometer measurements at 35°C yielded a viscosity of 3.7 × 10^−1^ Pa s, indicating that the glycerol is 99.5% pure. With an estimated temperature of 35°C, taking into consideration heating effects of the X-ray beam (Snell *et al.*, 2007 ▶), a viscosity of 2.3 × 10^−1^ Pa s for 76 µm particles and 4.3 × 10^−1^ Pa s for 42 µm particles was determined with RXT. Differences can be due to uncertainty in the temperature, with a 2.0°C lower temperature of the RXT measurements accounting for the disparity of the viscosity determined by the 42 µm particles. Errors in single particle tracking due to signal-to-noise and pixel size can be additional sources of error (Savin & Doyle, 2005 ▶; Martin *et al.*, 2002 ▶).

**Figure 4:**
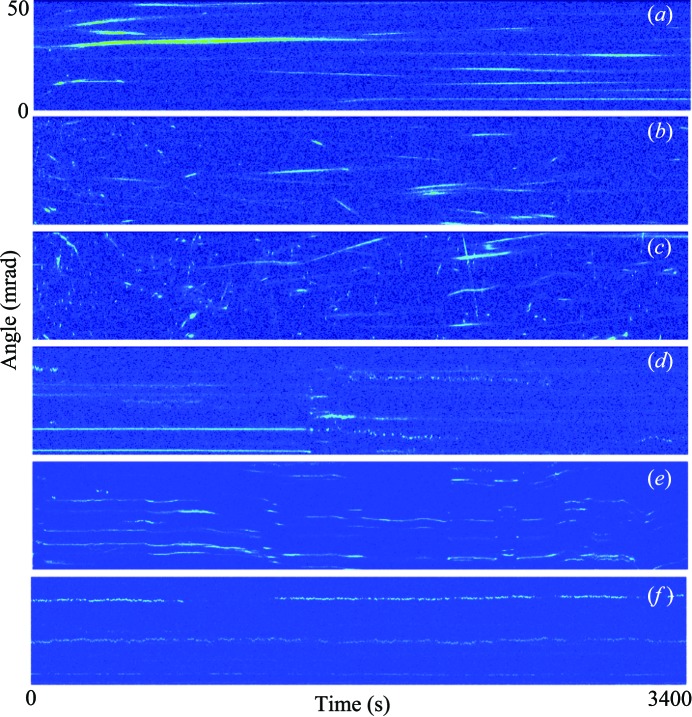
Rotational behaviors of alumina nanocrystals at temperatures above and below the melting point of decanoic acid: (*a*) −25.5°C, (*b*) 1.4°C, (*c*) 25.2°C, (*d*) and (*e*) 30.6°C – the melting point (*f*) slightly above the melting point. Average count rates are 3–4 photons per pixel in the Bragg peaks.

The alumina/decanoic system was studied over a range of temperatures, above and below the melting point of decanoic acid. In the solid phase, the alumina undergoes unidirectional rotational drift which increases in speed with higher temperatures (Figs. 4 ▶ *a*–*c*), while the liquid/gel phase shows stochastic behavior which varies little with temperature (Fig. 4 ▶ *f*). At the melting point, a variety of behaviors are observed, due to inhomogeneous heating/cooling in the macroscopic sample. Fig. 4 ▶(*d*) shows an abrupt transition from stationary motion to stochastic motion attributed to heat diffusion which results in a change of phase. Fig. 4 ▶(*e*) shows convective motion as a result of heating with many particles moving in concert, indicating that large domains of the liquid are rotating.

**Figure 5:**
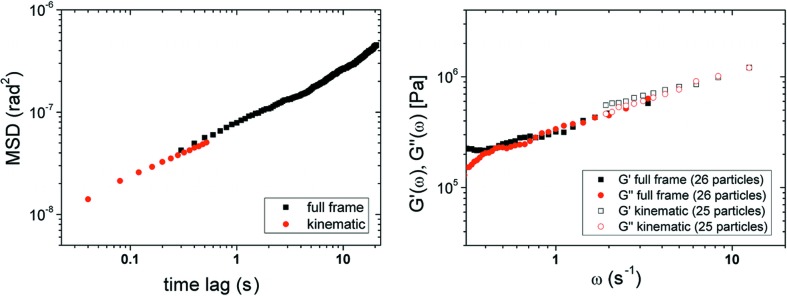
(*a*) The angular MSD *versus* time of 25 and 26 particles of 340 nm alumina in decanoic acid at 31°C with fast (kinematic – 25 particles) and slow (full frame – 26 particles) readout modes of the detector (Fig. S3), and (*b*) the corresponding *G*′(ω) and *G*′′(ω) obtained by a local power law expansion of the MSD.

Alumina/fatty acid gels have extremely high viscosities in the 10^5^ Pa s range (Bell *et al.*, 2005 ▶) which are thought to be due to the strong interparticle/intercluster forces involving the amphiphilic fatty acid which absorbs onto the surface. Calculated estimates for the interaction energy using van der Waals and steric interaction energies taken from Bell *et al.* (2005 ▶) and Bergstrom *et al.* (1992 ▶) put the interparticle energy at −18 kT, a relatively strong attractive gel. We observe that the majority of particles in diffraction had a step size of a few hundred microradians in 0.1 s^−1^, consistent with this high viscosity of alumina/fatty acid systems. Fig. 5 ▶(*a*) shows the angular MSD *versus* time average of 25 and 26 particles at 30°C with two different acquisition modes of the detector. The average of those particles shows subdiffusive behavior, defined as having a power law exponent of less than 1 on a log10 − log10 plot, indicative of non-Newtonian behavior.

Microrheology methodology can be used for the analysis of these subdiffusive data. The motion of the colloid particle which forms part of the gel scaffold, in our case the alumina nanocrystals, is determined by the viscoelastic properties of the gel itself, similar to a probe particle embedded in a surrounding medium (Mason & Weitz, 1995 ▶). The complex shear modulus G*(ω) = *G*′(ω) + *iG*′′(ω) describes a visco­elastic liquid with a real, storage component *G*′(ω) (the elastic modulus) and an imaginary, dissipative component *G*′′(ω) (the viscous modulus) (Ferry, 1980 ▶). G*(ω) can be experimentally determined from the MSD of the motion of an embedded tracer particle driven by thermal fluctuations (Mason & Weitz, 1995 ▶). The particle motion is described by a generalized Langevin equation with a time-dependent memory function η(*t*) related to the surrounding medium. Though the generalized Langevin equation is traditionally used to model the motion of an artificially embedded probe particle, we argue that it can also be applied to a piece of the medium itself under certain conditions in a similar way that the colloidal motion was used to determine viscoelastic properties for DLS data on a weak gel (Krall & Weitz, 1998 ▶). Our system can be described as a generalized Langevin equation with a viscoelastic probe particle. This viscoelastic probe particle would have internally viscous properties, allowing it to self-diffuse, and elastic properties, causing elastic deformation. In both cases, the motion would have additional contributions, indicating that the GSER would be inapplicable to the motion. However, we can easily eliminate the elastic deformation problem for our particles since our particles are hard spheres. Rotational self diffusion could be caused by the particle not being bound strongly to the gel network and diffusively rotating, but the binding energy of −18 kT indicates that the particle is strongly bound so there should not be a strong self-diffusion component. We thus conclude that the generalized Langevin equation taken for a segment of the gel network, *i.e* a single alumina particle, should be a good approximation. η(*t*) is related to the MSD *via* the velocity autocorrelation function using the Green Kubo formula, and also to the viscosity and G*(ω) (Ferry, 1980 ▶). The relation is given by the angular generalized Stokes Einstein relation (GSER).
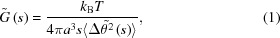
where *k*_B_ is the Boltzmann constant, *T* is temperature, *a* is particle radius, *s* is Laplace frequency and 〈Δ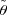 ^2^(*s*)〉 is angular MSD. Once the MSD is obtained, an inverse numerical Laplace transform is performed to obtain *G*′(ω) and *G*′′(ω). Mason *et al.* (1997 ▶) developed an alternative method using a local power law expansion to minimize truncation errors due to the finite extent of the data. SEM images show that the alumina crystals are cubic in shape (Fig. S2). Thus, the calculated rotational diffusion coefficient of a cube was used instead of that of a sphere (supporting notes) for the determination of *G*′(ω) and *G*′′(ω).

From the MSD *versus* time graph, the storage and dissipative modulus of the decanoic acid was determined (Bishop *et al.*, 2004 ▶). Our microscopic elastic modulus results are consistent with bulk studies for previous alumina/fatty acid experiments (Bell *et al.*, 2005 ▶; Bergstrom *et al.*, 1992 ▶) and the bulk elastic modulus of a colloidal gel of comparable particle concentration (Shih *et al.*, 1990 ▶; Pickrahn *et al.*, 2010 ▶; Schenker *et al.*, 2008 ▶).

## 5. Discussion   

Microstructuring of a material such as in a porous network or mesh-like gel gives rise to a length scale dependent visco­elasticity (Squires & Mason, 2010 ▶). Previous work to determine the viscoelastic properties using the colloid motion of added tracer particles were only sensitive to the low viscoelastic regime where probe particles diffuse freely within, or hop between, pores surrounded by the extended network (Pickrahn *et al.*, 2010 ▶; Gao & Kilfoil, 2007 ▶; Squires & Mason, 2010 ▶; Valentine *et al.*, 2001 ▶; Chen *et al.*, 2010 ▶). Studies on agarose gel have found that particles larger than the pore size, and thus coupled to the elastic network, have translational motion smaller than current resolution limits (Valentine *et al.*, 2001 ▶; Chen *et al.*, 2010 ▶). Similarly, in colloidal gel studies where the motion of the colloids are investigated, it is seen that the bulk elastic modulus increases with increasing fraction of ‘stationary’ colloids, indicating that they play the largest role in determining the bulk shear moduli (Shih *et al.*, 1990 ▶; Pickrahn *et al.*, 2010 ▶; Schenker *et al.*, 2008 ▶; Gao & Kilfoil, 2007 ▶; Dibble *et al.*, 2006 ▶; Guo *et al.*, 2011 ▶). RXT enables us to measure the local elastic network directly using the rotational MSD of the colloids. Agreement with bulk measurements of colloidal gel systems establishes that it is the network itself and not the voids formed by the gel that are being probed. We observe relatively homogeneous behavior of the alumina particles, indicating that the vast majority of particles are forming the colloidal gel with few, if any, freely diffusing within the intermediate free space.

Interestingly, we find that the viscous component of the gel is comparable to the elastic component of the rotational motion. Stiff colloidal gels do not allow for large translational diffusivity because the extended network itself is formed by inter-particle forces which causes *G*′(ω) to be much larger than *G*′′(ω). This confinement does not apply to rotational motion, however, and a particle can rotationally diffuse without compromising the structure of the colloidal gel unless there is site-specific bonding. There may be interdigitation or migration of surface layers during shear, although their contribution to bulk rheological measurements is determined to be small (Bell *et al.*, 2005 ▶). These effects, even if small, could contribute to the larger *G*′′(ω) values our experiment observes which is indicative of greater diffusivity for a single particle than would be expected for the bulk.

Typically the translational MSD for a system with visco­elasticity similar to our system would be characteristic of a sub-Å step size of the particle in 0.1 s, consistent with previous observations of ‘arrested’ particles. Sub-Å translational motion is unfortunately beyond the capability of any single particle tracking techniques developed so far and thus we cannot say that the translational diffusivity is expected to be lower than the rotational motion. It is, however, interesting to consider whether there is a fundamental difference between rotational and translational diffusivity in tightly bound systems such as gels. Recent studies with optical microscopy have found evidence that the translational and rotational motion of colloids decouple near the glass transition with the rotational motion not as hindered by steric jamming as the translational motion (Kim *et al.*, 2011 ▶). Those studies near the glass transition more directly probe the hydrodynamic interactions and local friction than the inter-particle forces that dominate our gel system. Given the many similarities between attractive colloidal glasses and colloidal gels, we hope that further investigations can explore the translational/rotational disparity for both.

We have used the method of rotational X-ray tracking to determine the rotational dynamics of crystalline colloids in a highly viscoelastic colloidal gel beyond the current limits of single particle techniques. We find agreement between microscopic and macroscopic elastic modulus for the first time and were able to determine previously undetermined values such as the rotational viscous response of the material. We have demonstrated the application of RXT to study the viscoelastic properties of Newtonian and non-Newtonian liquids, solids, and their phase transitions. The sensitivity of RXT enables investigation of theoretical predictions of rotational motion (Guerra *et al.*, 2010 ▶) which are currently inaccessible experimentally. Our observation of substantial rotational motion in solids and stiff gels which are widely thought of as stationary at the microscale suggests a whole new class of dynamical systems and materials. Technological advances in making core shell particles and nanocrystals embedded in traditional colloidal materials such as polystyrene or polymethyl methacrylate (PMMA) (Duguet *et al.*, 2000 ▶) could provide highly uniform and isotropic particles that can be directly compared with commonly studied colloids and used to study fundamentals of rotational motion.

## Supplementary Material

Supporting data and calculations. DOI: 10.1107/S2052252514006022/hf5259sup1.pdf

Video of data. DOI: 10.1107/S2052252514006022/hf5259sup2.avi
