# Supplementary material for: Brownian motion studies of viscoelastic colloidal gels by rotational single particle tracking
Source: IUCrJ. 2014 Apr 14;1(Pt 3):172–8. doi: 10.1107/S2052252514006022 (PMC4086434; doi:10.1107/S2052252514006022)
Supplement: Supplementary file 1 [file m-01-00172-sup1.pdf]

IUCrJ

Volume 1 (2014)

Supporting information for article:

Brownian motion studies of viscoelastic colloidal gels by rotational single particle tracking

Mengning Liang, Ross Harder and Ian K. Robinson

Figure S1 X-ray diffraction of dry alumina shows stationary behavior

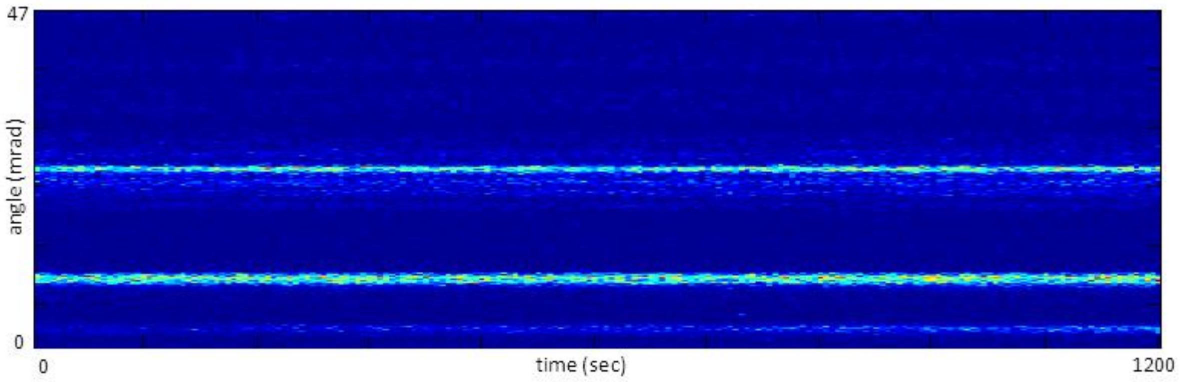

Figure S2 Scanning electron microscopy of 340nm alumina in decanoic acid. The particles do not aggregate due to the adsorption of decanoic acid onto the alumina particles. The smaller particles would appear with a much larger diffraction spot due to their smaller size and do not diffract strongly enough to track.

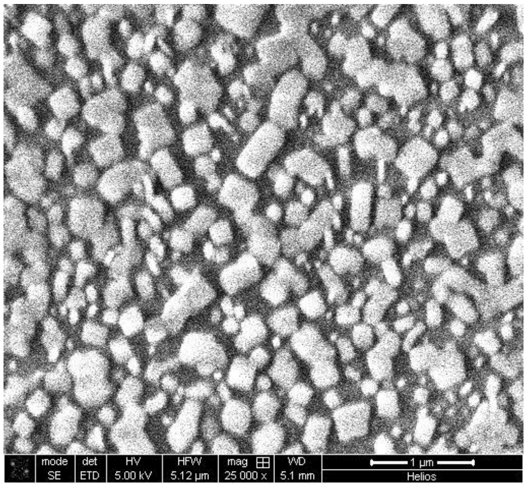

Figure S3 Two readout modes of the detector were used – a full-frame mode with slower readout is shown in the manuscript. The other mode is a kinetic fast readout mode where charge is shifted for a number of times before it is read out. Examples of such data, giving a time step of 0.02 sec is shown, each frame consists of 40 individual time steps, 26 pixels in width:

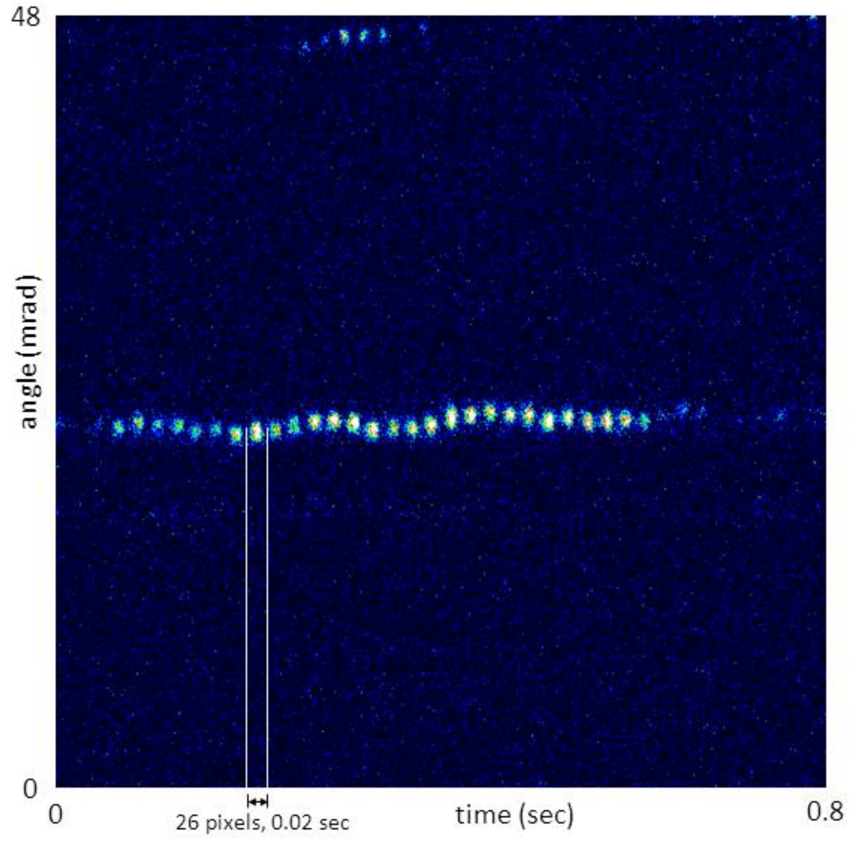

S1. Supporting Notes 1

Calculation of particle density within the illumination volume of the X-ray beam:
Wire scans of the focused X-ray beam measured a 1.85µmx1.9µm fwhm. The droplet has a diameter of ~5mm. Thus the total illuminated volume is ~$1.4 \times 10^{-14} m^3$.
The solid angle that the powder ring spans on the detector is 3.8×10^{-5} steradian. The (633) reflection (typically (104) in the equivalent hexagonal coordinates) and has a multiplicity of 12. One diffraction spot on the detector corresponds to 2.8×10^4 particles in the illuminated volume. Each cubic particle has a volume of $3.9 \times 10^{-20} m^3$.
There are on average 5 diffraction spots in the field of view of the detector at a single time step, leading to a volume packing of ~40%.

S2. Supporting Notes 2

Calculated diffusion coefficient of a cube:

(Ref 1) Gives the fundamental oligomeric structures used for the calculations of different shapes.

(Ref 2) Gives a correction to the former result. The diffusion coefficient for a cube of dimension 340nm is 0.48 times the diffusion coefficient of a circle with radius 170nm. The subunit is a sphere with radius 85nm.

1. Garcia Bernal, J. M. and De La Torre, J.G. Transport Properties of Oligomeric Subunit Structures. *Biopolymers* **20**, 129-139 (1981)
2. De La Torre, J.G., Lopez Martinez, M.C., and Garcia Molina, J.J. Approximate Methods for Calculating Rotational Diffusion Constants of Rigid Macromolecules. *Macromolecules* **20,** 3, (1987)
